# Annual Address of the President of the Erie County Medical Society

**Published:** 1857-02

**Authors:** Wm. Van Pelt

**Affiliations:** President


ART. V.—*Annual Address of the President of the Erie County Medical Society.* Delivered at the Annual Meeting, held in the city of Buffalo, Tuesday, January 13th, 1857. By WM. VAN PELT, M. D., President. Published by order of the Society.

*Gentlemen of Erie County Medical Society:*

A law of your society makes it imperative that your presidents shall read to you appropriate essays at the close of their terms of office. Agreeably to that regulation, I ask the indulgence of your patience while I invite your attention to the consideration of *the character of some of the objections often raised against the science of medicine.*

It has been said, "that the medical profession maintains peculiar relations to society." The peculiarity extends to the sciences toward which it looks for aid to enable its votaries to discharge the duties devolving upon them. The transcendental nature of the principles involved in the problems requiring solution, presents insurmountable obstacles, when precise knowledge is required. The medical art is expected to keep pace with the progress of the times. The study of nature as exhibited by the various phenomena incident to living bodies in health and disease, affords the only opportunities by which it can meet that expectation. Whenever it is desirable to bring questions within the reach of an accurate analysis, it is absolutely necessary to know the exact relations between the phenomena to be investigated, to have some manner of measurement that will admit of precise estimation. The whole of organic physics, it is, perhaps, needless to mention, is in that view far beyond the reach of human effort, on account of the great number and variability of the phenomena. It is for this reason that persons of respectable attainments in other departments of science are often disposed to ignore the claims of medicine to rank as a science. That this disposition exists, the experience of almost every person belonging to the profession will fully attest. The hundreds found in every community, who have acquired something of an idea of almost all things, and of none thoroughly, from the fashionable literature of the day, often express such opinions thus obtained. By such it is estimated to be a mark of intelligence to speak meanly of our venerable art. They reiterate sentiments coined by others. The study of specialties inclines the mind to place little confidence in results so variable as those which it presents. It is such persons, mainly, who have raised against it the objection "that the knowledge necessary to its successful application is so unattainable as not to be worth the time and trouble expended in its acquisition, that it is impossible to possess such power over the agencies of nature, by a knowledge of them, as to justify a reasonable confidence in measures applied with an intention to remove disease." This objection, if founded on truth, must deprive medicine of its claims to rank as a science. Its speciousness recommends it to the consideration of thousands of persons, who are necessarily ignorant of the proper character of medicine, and the basis upon which it rests its claims to such an examination. Medical men very often hear unjust reflections upon the character of it in respectable society; and they will continue to hear them so long as the popular ignorance remains concerning its true nature and character.

When we take into consideration the many thousands who have direct interests which require the destruction of popular confidence in regular medicine; that the press is diverted from its legitimate use, and is subservient to that interest, we are at no loss for reasons by which to account for this sentiment in relation to physic. It may be that in some degree the love of life and health has raised a general desire that sanitary measures, and the medical art, should possess a higher degree of knowledge and perfection than all others. The obscure conceptions generally entertained of the nature of the difficulties which involve most medical subjects, no doubt induces multitudes to withhold a proper respect. Medicine cannot be investigated with a mathematical precision, yet it possesses a vast amount of imperfect or partial knowledge which it is reasonable to perfect, preserve, and transmit to posterity. Notwithstanding it has not formulæ by which the laws of the ultimate forces and principles of nature can be known, still it is better to conceive them all as regulated by mathematical laws. Such a habit of regard is conservative, preventing the disposition to indulge in fanciful theories, the opprobrium of medicine. Besides, it is of the highest degree of probability, that any phenomenon traced to its ultimate cause, would show determinate circumstances, influences or powers as firmly fixed as they appear in gravitation. This partial knowledge makes it certain that there is some connection between phenomena; that there is a controlling cause why any effect is as it is, and not in any other manner. Although the boundary separating ignorance and knowledge, on most subjects, is not marked by monuments to guide the investigator, still it is as certain of this imperfect knowledge, as it is in regard to that which the universal testimony of mankind agree upon as absolutely certain. These hypercritical objectors would cast away all of this kind of knowledge, when but little reflection would show them that we are disposed to consider all results as having more or less probability; and we do not in any degree entertain the impression that they are fortuitous or unfixed. Although generally regarded as second in order of merit, it is relied upon in most of the business of the world, as in agriculture, commerce and mechanical pursuits. A large number of facts will exhibit a general law, if properly observed, recorded, and studied. It has not been generally supposed that this mothod of obtaining information has any utility in questions the elements of which involve the phenomena incident to vitality. Most events, before their happening or termination, are regarded as being invested with a sense of probability. This state applies to that mental impression which is produced by considering past events and the degree of future expectations. We always judge of the future, expecting in a degree that the same causes will have a continuous operation. So in making a diagnosis, we form our conclusions in regard to events that are to come, from the character of those that have appeared, and our knowledge of the general result in like or similar cases.

The system of statistics known as the "numerical method," is a rank higher of this second order of information, short of a rigid application of the theory of probabilities. It is believed that it makes known with tolerable accuracy, the tendencies of each disease to be influenced by age, sex, temperament, profession, &c. The theory of probabilities has been applied to but few medical subjects, other than life and health insurance. There are many questions to which I have no doubt, if it should be properly applied, it would serve to give more correct estimation of the value to be placed upon them. Whatever impression may be produced at the suggestion to treat all questions with a rigid accuracy, it nevertheless is the aim of our efforts; and there is no class of persons oftener required to form opinions, and act upon them, than physicians. How few, comparatively, of all those opinions but have been formed after a due estimation of the probabilities with which these subjects are surrounded. The opinions of different persons, on the same subject, are diversified by mental capacity, and education, as in other pursuits. And this diversity is adduced as proof of the inherent faultiness of the art; while observation shows that its errors are of the same character as those of other professions.

The nature of the questions requiring solution, involving, as it does, terms partially known, necessarily forces upon the mind a condition of uncertainty. If we express absolute certainty by 1 unit, the uncertainty may be expressed by a cypher (0). Then all degrees of certainty will be fractions of a unit. Sometimes a strict application of the theory may give results contrary to the general impressions; but when there is no agreement, we are inclined to adopt the results of the latter, numerically expressed. As an example of the application of the calculus, Brinkley's solution of Stokes' problem of contagion, affords a result so stupendous that the sense of certainty is perfect, to wit:

An epidemic prevails so severely that one person in seven dies. A family of twelve persons, in a particular district, is selected before the epidemic has visited it. What is the probability that eleven of the twelve shall take the disease, supposing the sickening of one of the family does not promote the sickening of another? Brinkley's answer is, 189,600,000 to 1 against the happening of such an event. It was suggested that local causes may reduce this ratio, but I think the problem includes infinite conditions, and Dr. Stokes improperly made the admission that such might be the case. An accurate record of 10,000 cases, treated according to the acknowledged rules of regular medication, and the record of another 10,000 not having had treatment, would, I have no doubt, exhibit by a comparison of results, the degree of power over the agencies of nature which medicine claims to possess. I am not aware that such a comparison has ever been instituted; but not making allowances to antecedent presumptions, a knowledge of the results derived from it, would furnish stronger arguments for, or against medication, than any that have been adduced. An extensive report of cases treated infinitesmally, would furnish an approximation to such information, but for the notorious abandonment of Hahnneman's minute doses, combined with the fact that the most successful practitioners of the system are persons who know something of regular practice. These circumstances would excite suspicion that the result would be vitiated. It is also assumed by another class of objectors, that all medication is mischievous in its effects. The falsity of this assumption is shown by daily observation: for the vast majority of medicines are salutary in cases requiring them: therefore the general probability is that they will prcve beneficial, whatever the result in a single case may be.

In 1000 cases a certain number of persons would recover without any such aid. The difference between that number, and the number treated, would show the power of medication. But to render the contest of opposite probabilities even so as to make the statement parallel to the assumption, we must suppose that no knowledge whatever is possessed of remedies, and that their exhibition is a matter of accident. Any degree of knowledge in relation to anything has its power, when it is used for the attainment of one object, and it thus turns the scales of chances toward certainty. Medical skeptics will always abound, and the character of them will be as various as mental organizations or habits of study, (if they are scientific persons,) shall induce, particularly if inclined to investigate limited fields of research

Many of this class seem to be overwhelmed by the complicated operations discovered in organic science; and they rush to an opposite extreme of disbelief because the *vital principle* is so incomprehensible. They forget that every department of science has incomprehensible ultimate principles. It makes no difference which of them we pursue, the same ultimate impassible barriers are found, and all objections raised against the medical art for any such reasons, are as absurd as they are against any other science. 'Tis true physiologists differ, but the character of those differences is precisely the same as are common to other subjects. Very much that has been written, has been with particular reference to the theological aspect of the question involved. To get rid of a vital principle, some have said that if we regard it as a peculiar manifestation of heat, mechanical power, or chemical affinity, our ideas of the operations of nature would be simplified; but the substituted theories are as inexplicable as before. When considering these subjects, we soon arrive at the horizon of human knowledge. Many metaphysical subjects which appear to have the best argument to sustain them, when adopted and fixed in the mind as true, produce such a departure from that simplicity and purity of character which so gracefully adorns the student of nature, that we have thus a proof of their unsoundness. Errors of reason and judgment would be of little moment did they not produce great aberrations in conduct. Experience and observation teaches that virtue and vice are the results of true or false opinions in regard to some fundamental principle. Every person entertains central sentiments around which he arranges all others in an order peculiar to the person. As might be expected, scientific medical skeptics fall the easy victims to some form of irregular practice. The regular medical practice possesses the confidence of every scientific person of the highest rank of generalizers, in all civilized countries. As a body they are united in their esteem. We should receive this as the highest testimony as well as compliment. Undoubtedly the reason why specialists are dissatisfied with medicine is, that depending as it must upon the circle of sciences for advancement, it must partake of their imperfections combined.

If we attempt to investigate the laws of light, heat, sound, gravitation, electric and magnetic repulsions, cohesion, chemical affinity, electrolysis, crystalization, capillarity, friction, or inertia, we find in each of them inexplicable forces. The laws of their operation, it is possible to imperfectly understand, and in each of them something is discovered that can be employed to contribute to the welfare of man, either adding to knowledge or physical comfort. The inclination to specialization, is found in medicine as in the mechanical arts. In fact those sciences most nearly connected to it may be considered in a degree specialties of it. In pursuing a specialty labor must become restricted in its field of operation; as the creed becomes more minutely investigated, calls for a farther division arise: thus investigation is extended. But this restriction of investigation and division of labor, while it produces a high degree of skill in an isolated direction, must, as a consequence, obscure the conceptions and weaken the power to judge of the importance of other fields of study.

An extended specialty becomes impracticable, when carried far beyond the adjoining areas, since there can be no connection or harmony.

A specialist, then, in other fields, must be a blunderer and a bigot.

Thus a chemist, pharmacean, or anatomist, may not necessarily understand the value to be placed upon a given theory, or medico-legal question.

A surveyor may make an accurate map of the streets and alleys of your city, but that ability imparts no information of the transactions by the wayside. Any attempts to form an opinion, or give a narrative of them, from such data, would justly excite ridicule. But an act of parallel absurdity is frequently exhibited by persons whose opinions would be received as authoritative on subjects which they understand. The natural proclivity to predicate opinions on subjects superficially investigated, while more caution is exercised on such as are pursued to gratify the peculiar taste of the individual, would be obvious, upon a little reflection. Such a disposition once realized, would, no doubt, furnish a salutary check upon hasty opinions. A knowledge of any subject cannot be obtained without at the same time learning something of others allied to it. An ambitious, presumptious character, or an enthusiasm in itself commendable, may also blind the mind and exalt a particular science above its rank. The generalities which lay above all specialties, can never be included in the consideration of it by the specialist of any sort, hence his opinions on general subjects of extended scope, must be of an inferior order. Then, again, generalization may be too enlarged, then it is indefinite, and useless, because impracticable; or generalization may be too circumscribed to include the points of special investigation, then the special is irreconcilable with the general, and discordance and loss of confidence is the result. The profession seems to require a great combination of these two dispositions. The specialist must be looked to to advance any science. Subjects of special study increase as knowledge is accumulated, the points of study becoming numerous, thus the form and extent of the science is established. When they are all extended, organized, it will stand a unit, a perfect science. Philosophical experiment has always been looked upon by the ignorant with feelings allied to superstition, hence the knaves and charlatans abounding employ these to wring from them the hard earned fruits of toil. Science and magic are confounded, and the expositor of knowledge is often esteemed to be of the same class.

The claims of chemistry are not ignored because its ultimate forces are inexplicable. Chemists entertain different views in regard to the theories of compound radicals, conjugation, homologies, and substitution. Each of these has contributed to the advancement of the knowledge of the art.

It is no objection against theories in general that they cannot be permanently established. Such an establishment would indicate that improvement in such departments, or rather on points within its sphere of consideration, is impossible on account of its perfection. The minds of most persons are perplexed to know the causes and phenomena in operation within observation. Theories are explanations of them, or believed to be, and are the result of systematic observation and reasoning. They are generally true for one or more terms in the series. But theories, like specialties, must mingle to form a general one, filling all the terms as knowledge is perfected. Differences in opinion in regard to the same subject, will often exist. Such differences may indicate independent investigation, or the impression produced by viewing the subject from different points. National theories reflect the characteristic animus peculiar to race. American medicine, although it has not a set of theories to sustain, has earned a name by exploding dogmas, by boldness of experiment and analysis, which has proved a salutary check upon the arbitrary enforcement of opinion so peculiar to governmental schools of other countries. In literature it no doubt lacks that classic neatness and accuracy that pertains to the profession in other countries. It is also charged against the profession, that the employment of the dead languages is done that physicians can the better impose upon the people. How much truth there may be to justify such a grave charge, I leave for each one to determine. Like all other useful agents, they may be perverted and a long incomprehensible record may too often serve as a fig leaf of ignorance.

Whilst I am fully sensible of their great usefulness, I am not fully convinced that if half the labor and time had been expended in making our language the one through which a perfect knowledge of medicine could be imparted to the American or English student, that much greater advancement could be made in the same length of time. If all names and expressions derived from the Greek or Latin languages were rendered in English, much of what is now considered as evincing refinement and culture, would appear as it really is, an evidence of a vitiated taste. Custom reconciles the most absurd practices; and many ridiculous fashions appeal so directly to pride, that it is difficult to view them in their just light.

As illustrative of the central thought of this essay, that the objections mainly against the art of medicine are of the same character as those against all other occupations or sciences, I will adduce several reports on medical subjects, made by the committees of the American Medical Association, and the sanitary reports of several cities. The subject of contagion or noncontagion, and its incidental connection with meteorology, I would particularly notice. Yellow fever and cholera are supposed to exemplify in their etiology, one or the other of these views. The circumstances, cases, and conditions relating to them are supposed to furnish facts from which may be determined the solution of the several questions entering into the discussion. These furnish very many points of disagreement. The influence of the atmosphere is supposed to be powerful in producing or aggravating diseases. In medical works it is enumerated as one of the most usual agents. The precise conditions necessary are never definitely stated, but are referred to in the most negligent manner. The failure of chemistry to give the necessary information by detecting the elements in the atmosphere, which are usually denominated *malaria*, leaves the subject open to the wildest conjecture. When we consider that the causes which produce small-pox, scarlatina, rubeola, or other infectious diseases, are unknown, also the relation they sustain to each other, it is not surprising that fanciful theories, and conflicting opinions, should exist. Lately the science of meteorology has been summoned to assist in making a solution of several interesting items in relation to this discussion. By it attempts have been made to find the precise conditions of the atmosphere, in places where severe epidemics were prevailing. Temperature, moisture, pressure of atmosphere, drying force, radiation, and the point of saturation, have been observed, calculated, and tabulated as the conditions determined. It is possible to ascertain them with a tolerable degree of exactness; to facilitate the reduction of observation, tables of the elastic force of vapor, and the actual amount of water in the form of invisible vapor have been determined, both by experiment and by calculation. The tables calculated by different persons differ, slightly, and on the whole are as nearly alike as the ability of different persons will permit.

The thermometer is relied upon to furnish the degrees of heat observed, and very properly, since it is by the power of this agent that many of the elements are changed: hence the necessity of the accurate observation of it. The name of the instrument (thermometer) would lead us to infer that it measured heat. Heat can be compared, but not measured. The increments of caloric at different points of the scale are very unequal. It is, perhaps, impossible to measure caloric. The difference of specific heat between the several degrees of the scale, has never been ascertained, because of the difficulty of making the experiments. Instruments cannot he employed without having a capacity for heat, and the thing that has it not is yet to be discovered. Direct experiment shows how much water a cubic foot of air can contain, at every degree of temperature of the scale. Now, assuming that an atom of caloric, and vapor, enter into the air, in a similar ratio, (I think there is no knowledge to the contrary) then it is possible to form something of an understanding of the value to be attached to the drying power. This condition, it appears to me, is not correctly estimated or appreciated by any observer with whom I am acquainted, either personally or by reputation.

For instance, if you draw at right angles to the scale of a thermometer, ordinates of the number of parts equal to the amount of water in grains, the air is capable of sustaining at that degree of scale; and if a curve is drawn through the extremity of these, the area contained between the scale and curve will very well represent the law by which vapor and heat pass into the atmosphere, supposing the surface to represent the capacity imparted. The drying power is usually reckoned by degrees, and gives, when calculated in that manner, very wrong information, sometimes. And at the best, our conception of the amount of it, cannot be perfect. The finding of the drying power should be done by estimating the area between the temperature of the point of saturation and the air temperature. It is a very easy experiment to prove that my theory of it agrees with the facts. An ounce of cotton moistened with 10 grains of water at temperature of 60°, with a drying power of 6°: and other at 80°, with the same amount of water and drying power, will dry much sooner at 80° than at 60°. The actual saturation and drying power are complimental to each other.

One of the most usual errors made by those who have given accounts of yellow fever and cholera epidemics, is the opinion that frequent showers indicate a wet air. The annual amount of rain in any country is one of the rudest observations: considered alone, it affords but little information concerning the amount of vapor. A great fault committed by those who seek in contagion alone the cause of disease, is, that nearly all of them give partial meteorological records, when the whole series should be given. One condition is sometimes compensated by another. For instance, a high dew-point is not always accompanied by a low drying power, consequently does not produce disease. An average high drying power is as productive of disease as a low one.

In the Transactions of the American Medical Association for 1856, is a report on yellow fever, by Dr. Cain, of Charleston; his report is a fair example of the misconceptions often entertained in relation to the precise meteorological conditions supposed to be present. He says: "the heat was greater after June than at any time since 1804. Hot as the weather was thermometrically, it was more so sensationally. The breezes were hot and parching, unrefreshing, for the reason they came from the land side. The systems of all exposed to such weather were, consequently, relaxed and enervated in a great degree, and several cases of *coup de soliel* occurred." He gives in connection tables of monthly rains, day heat and night heat, the average monthly dew points, but omits the solar radiation, drying power, relative humidity pressure, with other meteorological states. The direction from which the wind came, informs us somewhat in relation to the wanting data. It came loaded with hot vapor, solar radiation was great, since there was *coup de soliel;* the dew-point, by the record, was high in every month. Sensational heat is a different thing from actual heat. The report shows that he relied too much upon his sensations instead of good instruments.

Again: "The temperature of yellow fever summers, namely, 1849, 1852 and 1854, do not differ from those of 1850, 1851 and 1853. It was rather higher in the healthy ones than the others. Excessively wet summers have been healthy summers. In regard to the dew-point little need be said, it varies slightly in a few." Thus omitting to observe several of the items that are absolutely necessary to give a correct knowledge of the state of the atmosphere. His habit of affirming facts to have been in a certain way, and immediately contradicting the affirmation when speaking of some other points, renders it quite certain that he does not well understand the question he attempts to discuss. After having made an examination of the report of the sanitary commission of New Orleans, for 1850, he dismisses it in a summary manner as follows: "*The only condition uniformly present, was a high degree of heat; in all other respects they differed widely.*"

Unfortunately for his conclusion, he gave in the outset the dew-points of the four yellow fever months, namely, May, June, July, August, they were as follows: 68.67°, 72.58°, 76.70°, 77.29°: these, compared with those of New Orleans, of 1853, shows that two of them exceeded those, and the July average gave 4.57° higher. Here, then, we have a direct proof that heat was *not* the only similar condition.

Again: "Excessively wet summers,"—in other words, he means to say, that in the healthy summers it rained most—agreeably to the fact that a very little meteorological experience shows a drought is quite always attended with the greatest amount of vapor in the air. When this state continues long, the ground imparts its moisture, and the formation of dew is slight, because of diminished radiation.

In connection allow me to call your attention to the ratio of the drying power calculated, as I indicated, if you turn to Dr. Barton's Report, Table N·

| Average Temprature. | | Av. Drying Power. | Mortality. |
|---|---|---|---|
| January, | 47° | 16.44 | 581 |
| February, | 56.05° | 27.14 | 463 |
| March, | 62.33° | 36.13 | 456 |
| April, | 70.37° | 53.38 | 532 |
| May, | 73.82° | 48.75 | 671 |
| June, | 80.73° | 69.11 | 656 |
| July, | 79.88° | 62.78 | 2216 |
| August, | 81.25° | 48.81 | 6201 |
| September, | 76.23° | 45.46 | 1627 |
| October, | 60.81° | 48.44 | 674 |
| November, | 64.92° | 36.27 | 712 |
| December, | 53.06° | 27.07 | 844 |

If these numbers prove nothing, the coincidence is a singular one. The last four days of July gave a drying power very low; and 14 observations show perfect saturation in August.

Dr. La Roche, of Philadelphia, has just issued two large volumes on yellow fever. The great number of authors consulted, and the tediousness of detail, must overwhelm the reader with a high estimate of his patient industry. In looking them over it is manifest that he makes the same errors, meteorologically. He quotes old authors and their remarks of aerial conditions, when the subject was not so well understood, for evidence. The simple dew-point, which is so variously regarded, always comes in for a due share of attention, to the neglect of its coördinate observations necessary to allow of a comparison of the series. When it is known that he relies upon Professor Kirkpatrick's records, we know at once what value to place upon his conclusions. But few persons are aware of the tediousness and time attending the making of frequent observations. To find the dew-point experimentally, consumes too much time, therefore physicists have computed formulæ, by which it is found in a moment; unfortunately these differ, and observations are compared, in investigating these subjects, which will not admit of a comparison.

For example: my observations made Sept. 8 and 13, 1855, are as follows:

8th. Bar. 29.25 in.; air temp. 76.5°; evap. temp. 72.5°.
13th. " 29.1 "; " " 65.5°; " " 60.°

| | Sept. 8. | | Sept. 13. |
|---|---|---|---|
| Dew-point by Apjohn's formulæ, - - | 70.4° | and | 56.3° |
| " " corrected for altitude, - - | 70.55° | " | 56.5° |
| " " Glashen's factors, - - | 70.5° | " | 56.7° |
| " " Regnault, - - - - - | 70.1° | " | 56.6° |
| " " Greenwich, - - - | 70.8° | " | 56.5° |
| " " Engleman, - - - - | 70.9° | " | 56.06° |
| " " Barton's, - - - - | 70.8° | " | 55.9° |
| " " Kirkpatrick's, - - - - | 67.17° | " | 52.7° |

A glance will show how nearly the formulæ agree with the single exception of Kirkpatrick's. To satisfy myself, which of these was correct, I made a number of experimental observations of obtaining the actual point of saturation, and in no case did I differ one-tenth of a degree from the mean of the six.

We can place but little confidence in results reported, unless the formulæ are mentioned by which they were obtained. It needs but little investigation to show the necessity of all observers adopting the same method: one slightly wrong would be better than the present confusion.

Any attempt to prove Dr. Farr, Lee or Barton in error, in regard to their views of the epidemic character of the diseases in question, must, of consequence, fail, until more precision and faithfulness in making and reporting *the whole series of conditions* in connection with the other details. The thousands in the profession who make no observations, draw their conclusions in relation to these things, accordingly as some well written report may attract attention. The making of such observations, reducing them to a state to admit of an examination, requires more labor than one person can properly devote to such a work: it should be apportioned to several, the combined labors of the whole will produce results worthy of confidence. It would be very useful to organize a corp of observers in every city; but when the question arises as to remuneration, most of us are content to let such opportunities pass, and trust to chance, fashion, or accident, as to what opinions we shall entertain of these matters the next year, or through life. A more extended examination may show that a coincidence of conditions may originate the several materials or matters which produce what are called contagious affections. The time of the advent of small-pox, measles, scarlet fever, cholera, and yellow fever, are as accurately recorded, or rather the record is unanimous in its testimony of the periods as any matters of history. Upon many points in relation to them, many queries might be propounded as, Why did they not appear before? Is our record of their first appearance true? What becomes of these matters when they have been epidemic and have ceased? Will the locations which any of them has once visited, be liable to a recurrence of the disease, when a favorable state of the atmosphere or other surroundings will concur? If these materials do not lie latent wherever they are once deposited, why did we not have cholera before? Shall we ever get rid of it? All questions relating to these subjects, if they are ever answered correctly, will only be so after patient observation, comparison of results, and thorough examination. Theories will aid us in times past, and persons will continue to differ in opinion, for it is reasonable to expect that theories will not be in unison when matters cognizable to the senses fail to produce harmonious results, although capable of accurate measurement. The science of medicine is really censurable if it falls below the standard of perfection it might attain, by neglecting to apply the increased knowledge and improvements which other sciences acquire. It should not be expected to beat them, but it should pursue a parallel path. When physicists cannot agree upon the size of ultimate atoms, or the formulæ to represent the law of primary force of action, it appears very absurd to withhold a due respect to the medical art, because physiologists differ about the vital principle. The laws and nature of heat are imperfectly understood, and some characteristics have not been investigated, and require especial study. In nature, aggregation is entirely governed by it. It is the degree of it, which determines the forms of material existence. It must, then, determine the atomic constitution of each infectious material peculiar to those affections. If the existence of these should prove to be a myth, and all diseases, without exception, the product of a certain series of impressions upon the vital force, then very many views now entertained, will be discovered. A new era will dawn and many affections, now grievous affections to mend, will be preventable, by interrupting the series. Any advancement of knowledge in these directions, will eminently contribute to the comfort and longevity of the race.

Of all the difficult questions with which our art is beset, those of religion or infidelity has been most perplexing. The physiological student, when storing his mind with knowledge, is too often drinking in sentiments which will surely rise and impair that clear perception which might otherwise exist. A student of nature should follow truth wherever she leads, trustingly, like a child; if left in doubts and darkness let him continue until light appears. It would seem that the serpent had wound his slimy coils around the tree of knowledge, from the time of Adam until the present. The tempting fruit ever lying at the foot of man is poisoned at the core, and knowledge often proves to be a curse instead of a blessing.

No science, more than medicine, furnishes stronger proof that this world, to which we are attached, is peculiarly adapted to our wants and enjoyments, by supplying subjects to excite attention, and thus promote intellectual advancement; although surrounded with mysteries, enough of them lay within the grasp of thought to arouse the latent thirst for truth, and stir the plodding spirit to heavenly aspirations.

